# *CTLA4* promoter hypomethylation is a negative prognostic biomarker at initial diagnosis but predicts response and favorable outcome to anti-PD-1 based immunotherapy in clear cell renal cell carcinoma

**DOI:** 10.1136/jitc-2021-002949

**Published:** 2021-08-26

**Authors:** Niklas Klümper, Damian J Ralser, Romina Zarbl, Katrin Schlack, Andres Jan Schrader, Marc Rehlinghaus, Michèle J Hoffmann, Günter Niegisch, Annemarie Uhlig, Lutz Trojan, Julie Steinestel, Konrad Steinestel, Ralph M Wirtz, Danijel Sikic, Markus Eckstein, Glen Kristiansen, Marieta Toma, Michael Hölzel, Manuel Ritter, Sebastian Strieth, Jörg Ellinger, Dimo Dietrich

**Affiliations:** 1 Institute of Experimental Oncology, University Medical Center Bonn (UKB), Bonn, Germany; 2 Center for Integrated Oncology Aachen/Bonn/Cologne/Düsseldorf (CIO-ABCD), Bonn, Germany; 3 Department of Urology and Pediatric Urology, University Medical Center Bonn (UKB), Bonn, Germany; 4 Department of Obstetrics and Gynecology, University Medical Center Bonn (UKB), Bonn, Germany; 5 Department of Otorhinolaryngology, University Medical Center Bonn (UKB), Bonn, Germany; 6 Department of Urology, University Hospital Münster, Münster, Germany; 7 Department of Urology, University Hospital Düsseldorf, Medical Faculty, Düsseldorf, Germany; 8 Center for Integrated Oncology Aachen/Bonn/Cologne/Düsseldorf (CIO-ABCD), Düsseldorf, Germany; 9 Department of Urology, University Hospital Göttingen, Göttingen, Germany; 10 Department of Urology, University Hospital Augsburg, Augsburg, Germany; 11 Institute of Pathology and Molecular Pathology, Bundeswehrkrankenhaus Ulm, Ulm, Germany; 12 STRATIFYER Molecular Pathology GmbH, Cologne, Germany; 13 Institute of Pathology, St. Elisabeth Hospital, Cologne, Germany; 14 Comprehensive Cancer Center Erlangen-EMN (CCC ER-EMN), Erlangen, Germany; 15 Department of Urology and Pediatric Urology, University Hospital Erlangen, Friedrich-Alexander-University Erlangen-Nuremberg, Erlangen, Germany; 16 Institute of Pathology, University Hospital Erlangen, Friedrich-Alexander-University Erlangen-Nuremberg, Erlangen, Germany; 17 Institute of Pathology, University Medical Center Bonn (UKB), Bonn, Germany

**Keywords:** biomarkers, tumor, immunotherapy, urologic neoplasms, kidney neoplasms

## Abstract

**Background:**

In metastatic clear cell renal cell carcinoma (ccRCC), different combination therapies, each including anti-PD-1 immune checkpoint blockade (ICB), are applied as first-line treatment. Robust predictive biomarkers for rational upfront therapy decisions are lacking, although they are urgently needed. Recently, we showed that *CTLA4* promoter methylation predicts response to ICB in melanoma. Here, we aimed to investigate *CTLA4* methylation in ccRCC and its utility to serve as a predictive biomarker for anti-PD-1 based ICB in metastatic ccRCC.

**Methods:**

*CTLA4* methylation was analyzed with regard to transcriptional gene activity (mRNA expression), intratumoral immune cell composition, and clinical course in two ccRCC cohorts obtained from The Cancer Genome Atlas (TCGA cohort, n=533) and the University Hospital Bonn (UHB Non-ICB Cohort, n=116). In addition, *CTLA4* methylation as well as CD8^+^ T cell infiltrates and PD-L1 expression were evaluated in pre-treatment samples from a multicenter cohort (RCC-ICB Cohort, n=71). Patients included in the RCC-ICB Cohort were treated with either first line anti-PD-1 based combination therapy (n=25) or monotherapy post–tyrosine kinase inhibition in second line or later. Analyses were performed with regard to treatment response according to RECIST, progression-free survival (PFS), event-free survival (EFS), and overall survival (OS) following treatment initiation.

**Results:**

*CTLA4* promoter hypomethylation was significantly correlated with *CTLA4* mRNA expression, lymphocyte infiltration, and poor OS in both primary ccRCC cohorts (TCGA: HR 0.30 (95% CI 0.18 to 0.49), p<0.001; UHB Non-ICB: HR 0.35 (95% CI 0.16 to 0.75), p=0.007). In contrast, *CTLA4* promoter hypomethylation predicted response and, accordingly, favorable outcomes (PFS and OS) in patients with ICB-treated ccRCC, overcompensating the negative prognostic value of *CTLA4* hypomethylation at initial diagnosis. Moreover, in multivariable Cox regression, *CTLA4* promoter hypomethylation remained an independent predictor of improved outcome in ICB-treated ccRCC after co-adjustment of the International Metastatic Renal Cell Carcinoma Database Consortium score (HR 3.00 (95% CI 1.47 to 6.28), p=0.003).

**Conclusions:**

Our study suggests *CTLA4* methylation as a powerful predictive biomarker for immunotherapy response in metastatic RCC.

## Background

In the era of cancer immunotherapy, application of immune checkpoint blockade (ICB) targeting cytotoxic T lymphocyte–associated protein 4 (CTLA-4) and/or the programmed cell death 1 (PD-1/PD-L1) axis led to improved clinical outcomes in advanced clear cell renal cell carcinoma (ccRCC).^1–3^ Both combination of anti-PD-1 and anti-CTLA-4^4^ as well as combined anti-PD-1/PD-L1 plus a tyrosine kinase inhibitor (TKI)^5–8^ are currently applied as first-line therapy in metastatic ccRCC. As prospective clinical trials comparing these first-line therapies are still pending, both therapy combinations are currently considered equivalent in the intermediate and poor-risk groups defined by the International Metastatic Renal Cell Carcinoma Database Consortium (IMDC). In this context, a robust biomarker for an optimal upfront therapy decision and treatment sequencing in the clinical setting of metastatic ccRCC is missing.^1 9^

Tumor-intrinsic PD-L1 expression predicts response to anti-PD-1 ICB in various tumor entities, but in ccRCC it is of limited use and the European Association of Urology recommend not to consider this biomarker for patient stratification.^10–13^ Furthermore, a predictive biomarker that evaluates the effectiveness of an anti-PD-1 blockade is also of limited relevance in ccRCC, as the PD-1/PD-L1 immune axis is targeted in both first-line therapies as the current backbone of first-line ccRCC therapy. Of note, in CheckMate214, the study that ultimately led to the approval of ICB+ICB in ccRCC, only PD-L1 expression was evaluated regarding response rates,^4^ thereby excluding half of the biological mechanism of this therapy approach, precisely the blockade of the CTLA-4 immune checkpoint.^14^ A robust predictive biomarker for anti-CTLA-4 monotherapy is also currently lacking despite its high clinical relevance, as several new antibodies and probodies, which promise reduced off-tumor toxicity, are being developed and are already tested in clinical trials (eg, ClinicalTrials.gov Identifier: NCT03369223).^15^ In this context, we were recently able to provide strong evidence that the methylation status of the CTLA-4 encoding gene *CTLA4* predicts response to both anti-PD-1 and anti-CTLA-4 targeted ICB as well as anti-CTLA-4 monotherapy in patients with melanoma.^16 17^ In the present study, we therefore comprehensively investigated the promoter DNA methylation status of *CTLA4* in ccRCC with regard to transcriptional activity, clinicopathological parameters (including survival and response to ICB and TKI), immune cell infiltrates, and an interferon-γ signature. Understanding the epigenetic regulation of *CTLA4* in ccRCC is of major interest, as it might be promising as a predictive biomarker to enable a more rational therapeutic decision in favor or against ICB+ICB in patients with ccRCC in the age of individualized therapy.

## Methods

### Patient cohorts and clinical endpoints

#### TCGA cohort

Comprehensive methylation, expression, and immunogenomic data of the ccRCC TCGA dataset generated by *The Cancer Genome Atlas Research Network* (TCGA, http://cancergenome.nih.gov/) were used (n=533).^18–20^ Event-free survival (EFS) was previously recommended as a meaningful clinical endpoint for the ccRCC TCGA cohort and defined as progression of disease, local or distant recurrence, or death due to any cause.^21^

#### UHB Non-ICB Cohort

For validation purposes, a second previously described ccRCC cohort of patients treated at the University Medical Center Bonn (n=116) was included.^22^ According to the TCGA cohort, EFS was considered as a clinically meaningful endpoint in the UHB Non-ICB Cohort.

#### RCC-ICB Cohort

In addition, a multicenter ICB-treated RCC cohort was assembled (see table 1, n=71 also including n=4 non-ccRCC). The RCC-ICB Cohort included pre-treatment samples from patients who received either anti-PD-1 monotherapy second-line or later post-TKI (n=46) or first-line anti-PD-1 based combination therapy (n=25). Clinical endpoints were response to ICB according to RECIST V.1.1 and progression-free survival (PFS). PFS was defined as the time from ICB initiation until objective tumor progression or death. Overall survival (OS) was evaluated for all three cohorts.

**Table 1:** Patient characteristics of n=71 patients with metastatic (stage IV) RCC treated with anti-PD-1 ICB and association with PFS, response, and *CTLA4* promoter methylation

Characteristic	Total cohort (n*=*71)	PFS		*CTLA4* methylation		Response		
		HR (95% CI)	P value	Mean QMS (SD)	P value	ORR (n=20)	No ORR (n=51)	P value χ² test
Median age (range)	65 (44–79)	1.03 (0.99 to 1.06)	0.12					
Sex—no. (%)					0.52			0.58
Male	49 (69.9)	Ref group		73.8 (12.6)		14 (70.0)	35 (68.6)	
Female	22 (30.1)	0.81 (0.50 to 1.71)	0.81	71.4 (17.6)		6 (30.0)	16 (31.4)	
Sample origin—no. (%)					0.90			0.47
Primary	58 (81.7)	Ref group		73.1 (14.8)		17 (85.0)	41 (80.4)	
Distant metastasis	13 (13.8)	0.49 (0.22 to 1.13)	0.10	72.6 (12.1)		3 (15.0)	10 (19.9)	
RCC histology—no. (%)					**0.010**			0.31
ccRCC	67 (94.4)	Ref group		74.1 (12.8)		18 (90.0)	49 (96.1)	
Non-ccRCC	4 (5.6)	0.63 (0.15 to 2.61)	0.53	55.3 (25.5)		2 (10.0)	2 (3.9)	
ICB response—no. (%)					**0.030**			ND
Objective response*	20 (28.2)	0.06 (0.02 to 0.17)	**<0.001**	67.8 (18.5)		20 (100)	NA	
Stable disease	17 (23.9)	0.12 (0.05 to 0.27)	**<0.001**	72.4 (10.7)		NA	17 (33.3)	
Progressive disease	34 (47.9)	Ref group		79.8 (10.8)		NA	34 (66.7)	

P values comparing response refer to χ² test. Methylation levels between two or more groups were compared using Mann-Whitney *U* and Kruskal-Wallis tests, respectively. Cox proportional hazards were tested using Wald test.

*This category included patients with a complete response (n=4) and those with a partial response (n=16). Non-ccRCCs comprised two papillary, one chromophobe, and one medullary RCC. The origin of the included tissue of the distant metastases was lung (n=6), bone (n=4), and one each adrenal gland, skin, and gallbladder metastasis.

ccRCC, clear cell renal cell carcinoma; ICB, immune checkpoint blockade; NA, not applicable; ND, not determined; ORR, overall response rate; PFS, progression-free survival; QMS, Quantitative Methylation Score; RCC, renal cell carcinoma.

### Transcriptome data assembly

Log2-transformed RSEM (RNA-Seq by Expectation Maximization) RNA sequencing data (RNA-Seq v2) of *CTLA4*, interferon-γ signature and cytolytic activity genes (*IFNG*, *STAT1*, *STAT2*, *JAK2*, *IRF9*, *GZMA*, *GZMB*, *PRF1*) generated by Illumina HiSeq (Illumina, San Diego, CA, USA) were downloaded from the UCSC Xena browser (http://xena.ucsc.edu) (ccRCC n=533, normal adjacent tissue (NAT) n=72).

Comprehensive immunogenomic data on the composition of the tumor microenvironment and the interferon-γ signature response of the ccRCC TCGA cohort were obtained from Thorsson *et al* and implemented.^20^

### *CTLA4* promoter methylation analysis

**Figure 1:**
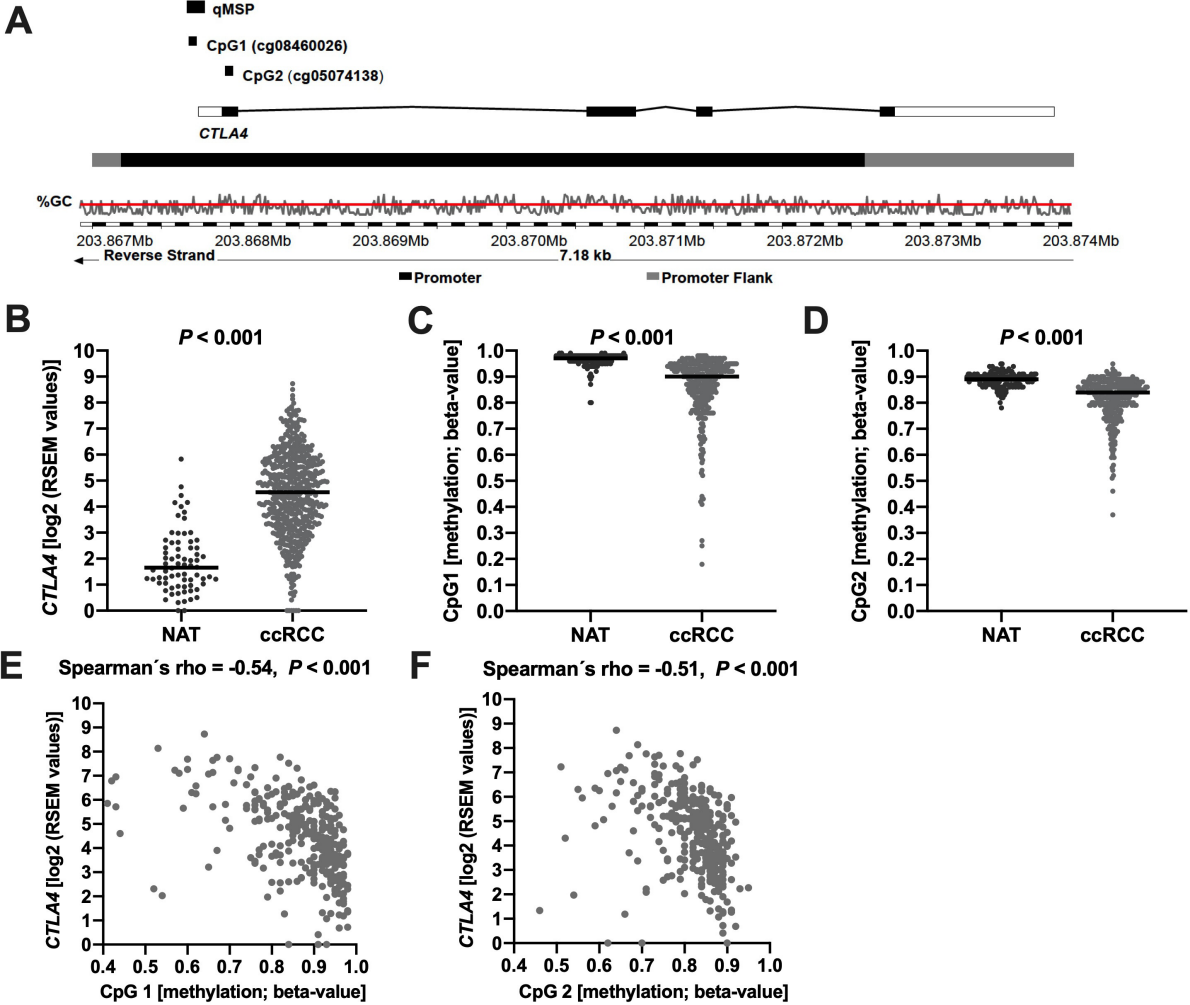
(A) Genomic organization of the *CTLA4* gene and target sites of the Human Methylation450 BeadChip (CpG1: cg08460026 and CpG2: cg05074138) and of the quantitative methylation-specific PCR (qMSP). The illustration (modified) was taken from Ensembl release 104 and is based on Genome Reference Consortium Human Build 38 patch release 13 (GRCh38.p13).^34^ (B–D) *CTLA4* mRNA expression and promoter methylation status of CpG1 and CpG2 in normal adjacent tissue (NAT) vs clear cell renal cell carcinoma (ccRCC). (E, F) Scatter plots representing *CTLA4* promoter methylation in relation to *CTLA4* mRNA expression.

The ccRCC TCGA cohort contained comprehensive methylation data from n=318 ccRCC and n=160 NAT samples. The CpG sites cg08460026 (CpG1) and cg05074138 (CpG2) within the *CTLA4* promoter were probed by beads from the Infinium HumanMethylation450 BeadChip (Illumina). The genomic organization of *CTLA4* is illustrated in figure 1A. *β* values, estimating the ratio of intensities between methylated and unmethylated alleles, were used for analyses.

In the UHB Non-ICB Cohort (n=116) and the RCC-ICB Cohort (n=71), we used a quantitative methylation-specific PCR (qMSP) assay in order to determine the methylation level of CpG1 within the *CTLA4* promoter. The qMSP assay contained primers that amplify methylation—unspecifically a 73 bp amplicon (forward primer: attcaattaaatacttaaaattatcttttc, reverse primer: tatatatgtgtatatatagaaggtatttg). The assay included two hydrolysis probes that specifically and competitively hybridize to methylated and unmethylated *CTLA4* sequences, respectively (methylated: 6-Fam-cccacgacttcctttctcgtaaa-BHQ-1, unmethylated: HEX-acccacaacttcctttctcataaaacc-BHQ-1). The assay probes CpG1 and an adjacent CpG site (genomic target sequence: CGGCTTCCTTTCTCG). We calculated Quantitative Methylation Scores (QMS) using the formula: QMS=100/(1+2^∧^(CT_methylated_−CT_unmethylated_)).^23 24^ We used a PCR buffer composition as described earlier^25^ and ran the PCR for 20 min at 95°C and 40 cycles with 15 s at 95°C, 15 s at 55°C, and 60 s at 52°C using a 7900HT Fast Real-Time PCR system (Applied Biosystems, Waltham, MA, USA).

### Immunohistochemistry

Immune cell infiltrate scores of CD4^+^ and CD8^+^ T cells and pan-leukocytes (CD45^+^) as quantified via immunohistochemistry (IHC) on whole slides were included from our previous work (UHB Non-ICB Cohort).^22^ CD8^+^ T cell infiltration in tumors from the multicenter RCC-ICB Cohort was evaluated accordingly.^22^ PD-L1 IHC 22C3 pharmDx (Agilent Technologies, Santa Clara, CA, USA) was used for the assessment of PD-L1 Combined Positive Score (CPS) for the RCC-ICB Cohort following the manufacturer’s instructions.

### Statistics

Microsoft Excel, GraphPad Prism, and SPSS V.25 were used for statistical analyses. Non-parametric Spearman’s *ρ* correlation coefficients were calculated. Group comparisons were made using parametric two-sided Student’s t-test or nonparametric Mann-Whitney U or Kruskal-Wallis (>2 groups) test. Survival analyses of median dichotomized variables were performed using the log-rank test and visualized via Kaplan-Meier plots. Continuous log2-transformed variables were used for Cox proportional HR analyses with specified 95% CI.

## Results

### *CTLA4* promoter is hypomethylated in ccRCC compared with normal adjacent renal tissue (NAT)

We investigated methylation of two CpG sites, referred to as CpG1 and CpG2, located in the central promoter region of *CTLA4* (figure 1A). Interestingly, both evaluated CpG sites showed significant hypomethylation in ccRCC compared with NAT (p<0.001) and, inversely, *CTLA4* mRNA expression was increased in ccRCC versus NAT (figure 1B–D). In addition, both analyzed CpG sites showed a high degree of co-methylation (Spearman’s *ρ*=0.68, p<0.001).

### *CTLA4* transcriptional activity is associated with its promoter methylation

Next, we aimed to analyze to what extent the transcriptional activity of the *CTLA4* gene is associated with the methylation status of its promoter region. In ccRCC, hypomethylation of both CpG sites within the promoter was inversely correlated with *CTLA4* mRNA expression with significant Spearman’s correlation coefficients: CpG1 *ρ*=−0.54; CpG2 *ρ*=−0.51 (both p<0.001, figure 1E, F). Thus, the transcriptional activity of the *CTLA4* gene strongly depends on its promoter methylation. In NAT, no significant correlation between the *CTLA4* promoter methylation and its mRNA expression was evident, which might be due to a low sample size (n=24) or indicates a tumor-specific methylation pattern.

### *CTLA4* promoter hypomethylation and *CTLA4* mRNA expression are associated with distinct immune cell infiltration and an interferon-γ expression signature

**Figure 2:**
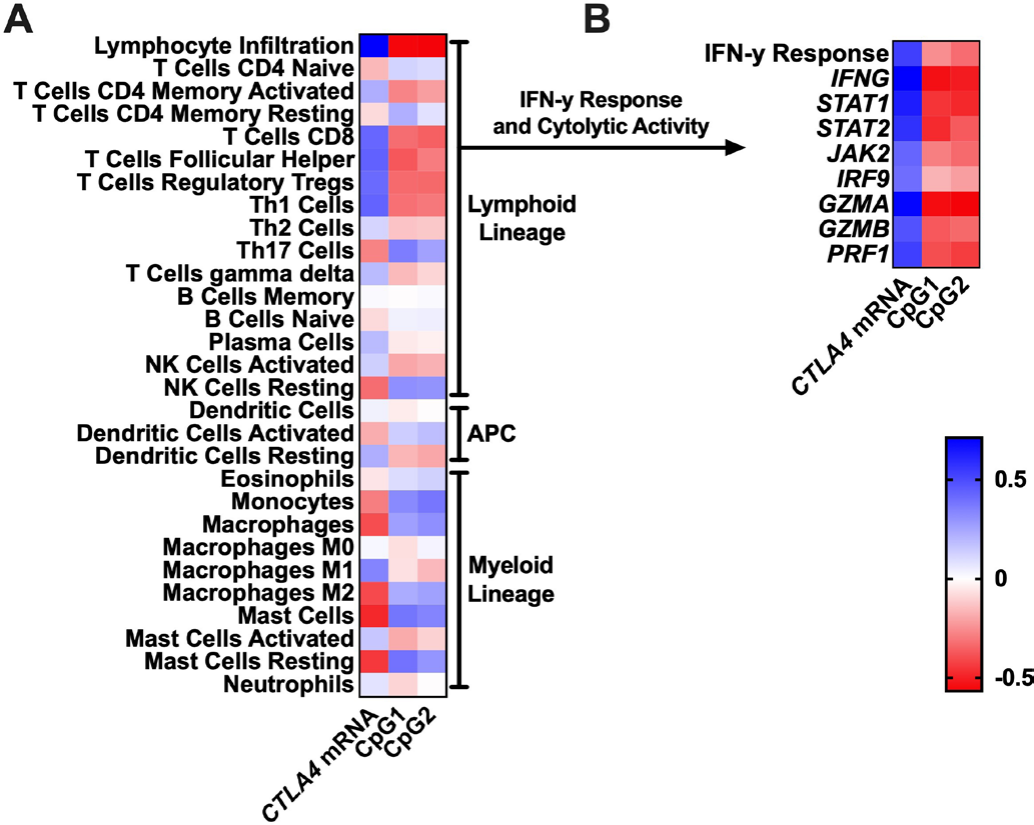
Correlation heatmaps visualize Spearman’s *ρ* correlation coefficients of both the *CTLA4* mRNA expression and the promoter methylation status (CpG1+2) with respect to the intratumoral immune cell composition (A), the IFN-γ response, and cytolytic activity (B), respectively. APC, antigen-presenting cell.

The tumor microenvironment is a complex assembly of different immunological cell types. Since tumor immunogenicity is an essential component for the success of ICB,^14^ we next aimed to investigate to what extent *CTLA4* methylation status is associated with the intratumoral immune cell composition. *CTLA4* promoter methylation, as well as mRNA expression, significantly correlated with the overall lymphocyte infiltration score (figure 2A). Considering the different subtypes of the lymphoid lineage, it is noteworthy that especially signatures of CD8^+^ T cells, T follicular helper cells, regulatory T cells (Treg), and Th1 cells were associated with upregulated *CTLA4* mRNA expression and concurrent *CTLA4* promoter hypomethylation. In contrast, signatures of myeloid infiltration, especially monocytes and macrophages (in particular M2 macrophages), was correlated with low *CTLA4* mRNA expression and accompanying promoter hypermethylation. Next, we evaluated the relationship between *CTLA4* promoter methylation with the interferon-γ (IFN-γ) response signature and the cytolytic activity (*GZMA*, *GZMB*, *PRF1*), which are both well known to influence ICB efficacy.^11 26^ Of note, both *CTLA4* promoter hypomethylation and mRNA expression were strongly associated with increased IFN-y signatures and cytolytic activity (figure 2B).

### *CTLA4* promoter hypomethylation is associated with an unfavorable clinical course in ccRCC

**Figure 3:**
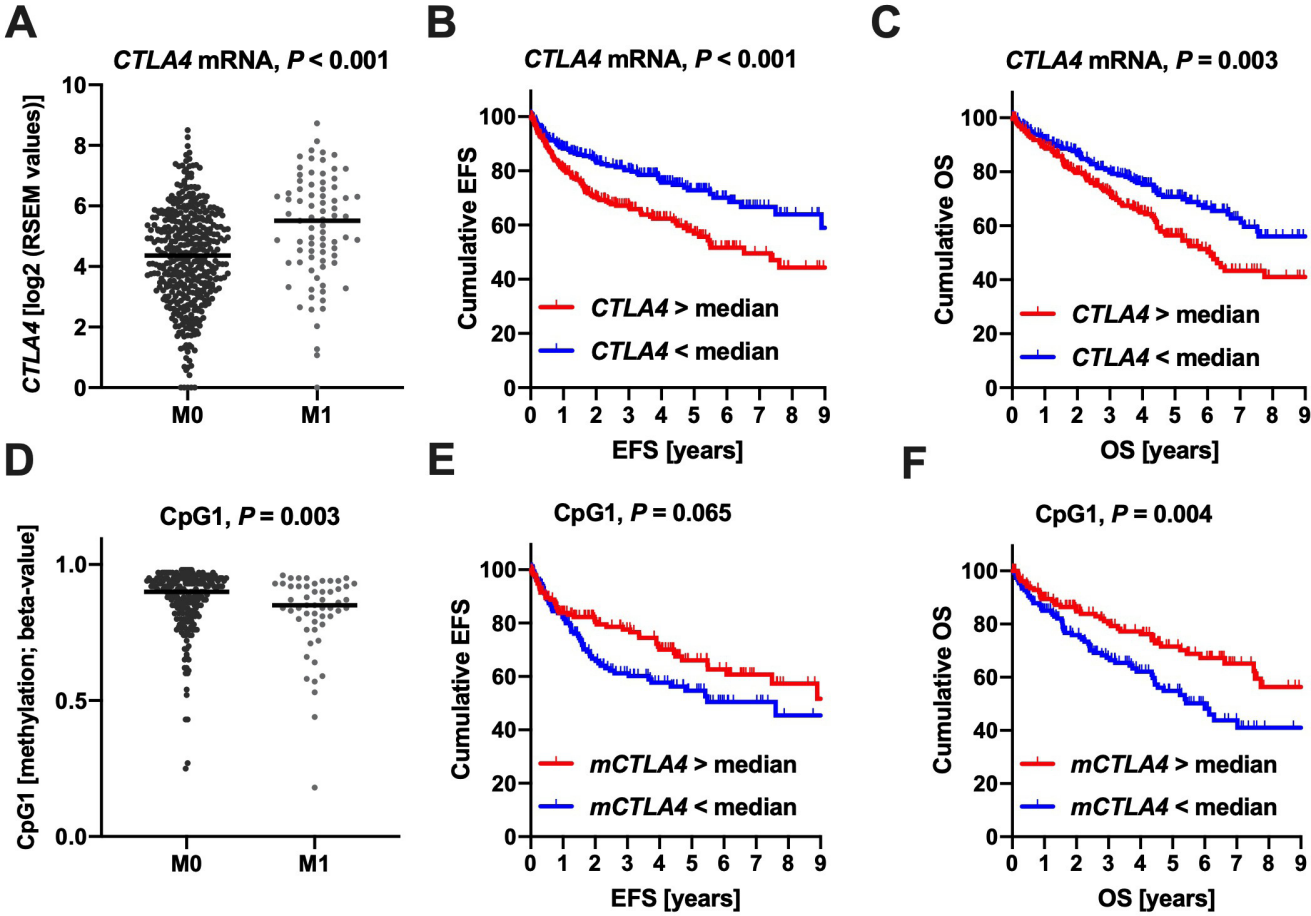
Association of the *CTLA4* mRNA expression and promoter methylation of CpG1 with respect to the metastatic status (M stage, A, D) and the clinical endpoints event-free survival (EFS, B, E) and overall survival (OS, C, F) in the ccRCC TCGA cohort are depicted. ccRCC, clear cell renal cell carcinoma.

Next, we evaluated to what extent *CTLA4* promoter methylation and *CTLA4* transcriptional activity were associated with metastatic spread, the crucial step in ccRCC progression, and clinical outcomes. *CTLA4* overexpression and concurring hypomethylation in primary ccRCC (TCGA cohort) were strongly associated with metastatic spread (figure 3A, D). After dichotomization of the cohort by the median *CTLA4* mRNA expression, the overexpressing subgroup exhibited a worse clinical course regarding both event-free (EFS, HR 1.23 (95% CI 1.12 to 1.36), p<0.001) and overall survival (OS, HR 1.25 (95% CI 1.14 to 1.38), p<0.001, figure 3B, C). In accordance, promoter hypomethylation of CpG1 was associated with unfavorable EFS (HR 0.36 (95% CI 0.22 to 0.60), p<0.001) and OS (HR 0.30 (95% CI 0.18 to 0.49), p<0.001, figure 3E, F). The methylation status of CpG2 showed only a trend and no significant association with outcome (EFS: HR 0.20 (95% CI 0.03 to 1.57), p=0.13; OS: HR 0.15 (95% CI 0.02 to 1.13), p=0.065).

**Figure 4:**
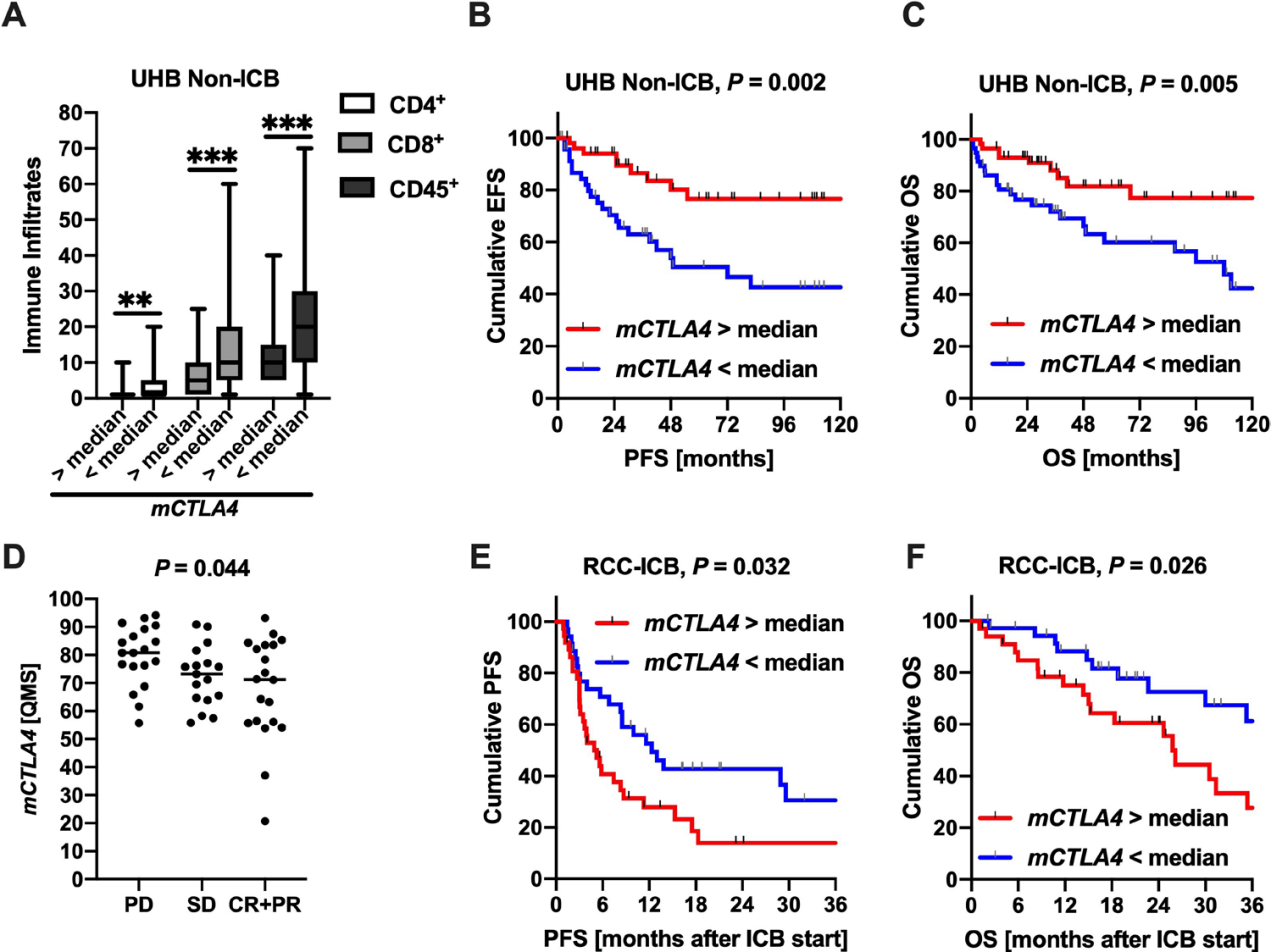
(A) *CTLA4* promoter hypomethylation is associated with high lymphocyte infiltration, especially CD8^+^ T cells. (B+C) *CTLA4* promoter hypomethylation was associated with unfavorable event-free (EFS) and overall survival (OS). (D) In pre-treatment RCC samples, *CTLA4* promoter hypomethylation predicts immune checkpoint blockade (ICB)–treatment response and is associated with prolonged progression-free survival (PFS) after ICB-treatment initiation (E) and favorable OS (F).

To validate the aforementioned results in an independent cohort, we evaluated *CTLA4* promoter methylation in our UHB Non-ICB Cohort (n=116) using a quantitative methylation-specific PCR assay. The qMSP targets CpG1 and one adjacent CpG site located 13 bp upstream from CpG1 (figure 1A). Of note, *CTLA4* promoter hypomethylation was correlated with an enriched immune cell infiltration pattern (IHC for CD4^+^, CD8^+^ T cells and pan-leukocytes (CD45^+^)), which validates the aforementioned results that were based on immunogenomic RNA-Seq signatures (figure 4A). Confirming our results from the TCGA cohort, *CTLA4* hypomethylation in primary ccRCC tissue at initial diagnosis was significantly associated with unfavorable EFS (HR 0.36 (95% CI 0.17 to 0.78), p<0.010) and OS (HR 0.35 (95% CI 0.16 to 0.75), p=0.007) in the UHB Non-ICB Cohort (figure 4B, C).

### *CTLA4* promoter hypomethylation predicts response and outcome in metastatic RCC treated with anti-PD-1 immunotherapy

Since *CTLA4* promoter hypomethylation is associated with an enhanced immune infiltrate in ccRCC, assessment of *CTLA4* methylation status in treatment-naïve tissue samples prior to initiation of ICB therapy may have predictive value for ICB-treatment success. To evaluate the potential predictive value of *CTLA4* methylation prior to immunotherapy, we have assembled a multicenter ICB treated cohort (RCC-ICB Cohort, n=71) of patients treated at German tertiary referral centers. As shown in primary ccRCC earlier, *CTLA4* hypomethylation again correlated strongly with CD8^+^ T cell infiltration in the RCC-ICB cohort (Spearman’s *ρ*=−0.44, p<0.001).

Of note, *CTLA4* promoter hypomethylation in pre-treatment RCC samples predicted ICB-treatment response (figure 4D). In concordance with the better response of *CTLA4* hypomethylated tumors to immunotherapy, patients experienced a prolonged PFS and OS after ICB treatment initiation (PFS: HR 1.94 (95% CI 1.09 to 3.44), p=0.024; OS: HR 2.14 (95% CI 1.01 to 4.57), p=0.048; figure 4E, F). This finding is of particular importance because, in contrast, *CTLA4* hypomethylation at initial diagnosis was associated with worse survival. Therefore, the positive predictive value of *CTLA4* hypomethylation exceeded the negative prognostic value at initial diagnosis (TCGA, UHB Non-ICB). For a subgroup of n=44 patients in the RCC-ICB Cohort, response data on prior or subsequent TKI were available. In contrast to its predictive value on immunotherapy, *CTLA4* methylation status did not predict TKI-treatment success and outcome.

Of note, intratumoral PD-L1 expression (cut-off CPS>1) had no predictive value in our multicenter RCC-ICB cohort (PFS: HR 1.46 (95% CI 0.78 to 2.74), p=0.24). Furthermore, in multivariate Cox regression, *CTLA4* promoter hypomethylation remained an independent predictor of improved outcome following ICB-treatment initiation after co-adjusting the IMDC risk score (HR 3.00 (95% CI 1.47 to 6.28), p=0.003).

## Discussion

An epigenetic regulation of the *CTLA4* gene via DNA methylation has already been observed in melanoma.^16^ Interestingly, and of particular clinical interest, the *CTLA4* methylation status exhibited a predictive value in patients with melanoma treated with anti-PD-1 plus anti-CTLA-4 immune checkpoint therapy.^16^ In the current clinical situation for metastatic ccRCC, a robust predictive biomarker for this particular ICB combination therapy, anti-PD-1 plus anti-CTLA-4, is urgently needed as the combination of anti-PD-1/PD-L1 plus TKI is currently considered equivalent and comparative studies are still pending.^1 4–6 9^ In this study, we therefore comprehensively investigated *CTLA4* promoter methylation with regard to transcriptional activity, clinicopathological parameters, and the intratumoral microenvironment in ccRCC tissue. Of note, we observed a strong correlation between the transcriptional activity of *CTLA4* and its promoter methylation status in ccRCC. Moreover, *CTLA4* promoter methylation and its mRNA expression showed a significant association with the composition of the ccRCC tumor microenvironment: *CTLA4* overexpression and concomitant promoter hypomethylation were associated with particularly high lymphocyte infiltration and an increased interferon-γ signature as well as cytolytic activity. *CTLA4* hypomethylation thus appears to be a robust surrogate biomarker for an enriched tumor microenvironment. Further, an unfavorable clinical course was evident in primary RCC with hypomethylated *CTLA4* promoter and overexpression, respectively. These findings are in line with the literature describing increased immune cell infiltration and immune checkpoint expression in RCC as a negative prognostic marker.^26^

The transcriptomic and methylation data in the ccRCC TCGA dataset were obtained from whole tumor tissue samples of patients with ccRCC receiving nephrectomy^18 19^ and are therefore based on the genomic signature of the tumor and its microenvironment, including tumor cells, stroma, infiltrating immune cells, and tumor-associated fibroblasts.^27^ The complexity of epigenetics is highlighted by widespread tissue-specific and cell type–specific methylation patterns in diverse biological processes;^28^ however, the characterization of an existing cell line–specific epigenetic regulation of *CTLA4* via DNA methylation patterns was not the focus of our study. The aim of our study was to investigate a predictive and whole-tissue based easy-to-implement biomarker for RCC, and excitingly, *CTLA4* promoter methylation seems to have cancer-independent predictive potential for ICB response in melanoma and RCC.^16 17^

As a chemically stable epigenetic modification that is not as dynamic as mRNA or protein expression, DNA methylation patterns represent particularly attractive biomarkers.^29^ Furthermore, the fact that quantitative and investigator-independent measurement of DNA methylation is even possible in small samples (microdissected cells, liquid biopsies, circulating tumor cells) is a major advantage from the diagnostic point of view.^30^ ^31^ Basing the data on *CTLA4* DNA methylation shown here and the data on *CTLA4* methylation in melanoma^16 17^ strengthen the rationale to test this particular methylation biomarker in clinical trials. In the present study, we have analyzed uncalibrated quantitative methylation levels by means of *ß* values (Illumina Infinium Technology) and QMS values (qMSP), respectively. These levels, however, do not necessarily reflect true percentage methylation levels. In order to determine percentage methylation, for example, for the transfer of clinically relevant cut-offs to different platforms and assay technologies, absolute methodologies, that is, bisulfite clone sequencing, could be applied.

The *CTLA4* promoter hypomethylated ccRCC subgroup was characterized by enhanced immune cell infiltration, in particular, CD8^+^ T cell infiltration indicating these tumors as immunologically “hot tumors". Thus, we asked the question whether the *CTLA4* methylation status in treatment-naïve tissue samples prior to initiation of ICB therapy has predictive value to immunotherapy in RCC. Of note, in our multicenter RCC-ICB cohort, *CTLA4* promoter hypomethylation predicted ICB treatment success, which also translated into prolonged PFS and OS after ICB treatment initiation, thereby counteracting its negative prognostic value in primary ccRCC at initial diagnosis. *CTLA4* methylation status was not associated with TKI response, highlighting that *CTLA4* methylation appears to be predictive for immunotherapy only. At initial diagnosis (TCGA and UHB Non-ICB Cohorts), *CTLA4* promoter hypomethylation was a negative prognostic biomarker and associated with poor outcome, whereas in metastatic stage prior immunotherapy, it was a favorable biomarker. This is most likely due to its predictive value since the high response to ICB overcompensated the negative prognostic value at initial diagnosis. A similar phenomenon has already been described for melanoma. PD-L1 upregulation is associated with an aggressive subset of melanomas with unfavorable outcome at initial diagnosis but has predictive value for ICB response.^32 33^ Thus, negative prognostic biomarkers at baseline with strong predictive value for immunotherapy response can overcome their initial negative prognostic value in advanced disease stages. This highlights the potential of *CTLA4* methylation as a promising predictive biomarker prior to ICB-treatment initiation in RCC, which has already been suggested for melanoma.^16 17^

In the current clinical setting of metastatic RCC with multiple first-line therapies, essentially either ICB+TKI or ICB+ICB, there is a tremendous clinical need for robust predictive biomarkers for rational upfront therapy selection, but despite significant efforts, no biomarker that can be easily implemented into clinical practice is available. PD-L1 expression is the only broadly used predictive biomarker, but in ccRCC it is of limited clinical use.^10–13^ However, patients with ≥1% PD-L1 expression seem to benefit particularly from intensified immunotherapy with nivolumab plus ipilimumab.^3^ In our multicenter RCC-ICB Cohort, *CTLA4* promoter hypomethylation outperformed PD-L1 CPS, which had no significant predictive value in our cohort. Thus, it remains to be prospectively elucidated whether the predictive potential of *CTLA4* promoter methylation status will lead to an improved stratification for rational upfront treatment decisions for either ICB+TKI or ICB+ICB.

The main limitations of our study are the retrospective design, the relative small sample size of our RCC-ICB cohort, the heterogeneity of included patients regarding histology (clear-cell and non-clear-cell RCC included), sample origin (primary tumor and distant metastases), and pre-treatment. In order to establish a robust biomarker in this clinical setting, prospective studies are needed to determine the clinical performance of *CTLA4* promoter hypomethylation as a predictive biomarker for ICB in patients with ccRCC.

## Conclusion

In ccRCC, the important immune checkpoint CTLA-4 is epigenetically regulated by promoter DNA methylation. *CTLA4* promoter hypomethylation is a strong biomarker for poor prognosis in patients with ccRCC at initial diagnosis. In contrast, *CTLA4* promoter hypomethylation predicted response and favorable outcome to immunotherapy in our multicenter ICB-treated RCC cohort. Thus, it represents a promising candidate for the urgently needed predictive biomarker for optimal upfront treatment decision in metastatic ccRCC.

## Data Availability

Data are available on reasonable request. The results shown here are based on data generated by The Cancer Genome Atlas project (TCGA, http://cancergenome.nih.gov/) (18). Data based on the UHB Non-ICB cohort and the RCC-ICB cohort are available on reasonable request.
